# Perception and classification of emotions in nonsense speech: Humans versus machines

**DOI:** 10.1371/journal.pone.0281079

**Published:** 2023-01-30

**Authors:** Emilia Parada-Cabaleiro, Anton Batliner, Maximilian Schmitt, Markus Schedl, Giovanni Costantini, Björn Schuller

**Affiliations:** 1 Institute of Computational Perception, Johannes Kepler University Linz, Linz, Austria; 2 Human-centered AI Group, Linz Institute of Technology (LIT), Linz, Austria; 3 Chair of Embedded Intelligence for Health Care and Wellbeing, University of Augsburg, Augsburg, Germany; 4 Department of Electronic Engineering, University of Rome Tor Vergata, Rome, Italy; 5 GLAM—Group on Language, Audio & Music, Imperial College London, London, United Kindom; Indian Institute of Technology Patna, INDIA

## Abstract

This article contributes to a more adequate modelling of emotions encoded in speech, by addressing four fallacies prevalent in traditional affective computing: First, studies concentrate on few emotions and disregard all other ones (‘closed world’). Second, studies use clean (lab) data or real-life ones but do not compare clean and noisy data in a comparable setting (‘clean world’). Third, machine learning approaches need large amounts of data; however, their performance has not yet been assessed by systematically comparing different approaches and different sizes of databases (‘small world’). Fourth, although human annotations of emotion constitute the basis for automatic classification, human perception and machine classification have not yet been compared on a strict basis (‘one world’). Finally, we deal with the intrinsic ambiguities of emotions by interpreting the confusions between categories (‘fuzzy world’). We use acted nonsense speech from the GEMEP corpus, emotional ‘distractors’ as categories not entailed in the test set, real-life noises that mask the clear recordings, and different sizes of the training set for machine learning. We show that machine learning based on state-of-the-art feature representations (wav2vec2) is able to mirror the main emotional categories (‘pillars’) present in perceptual emotional constellations even in degradated acoustic conditions.

## Introduction

**Fig 1:**
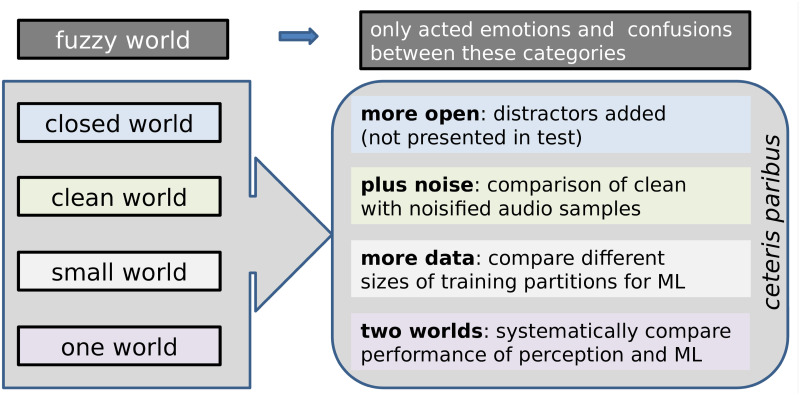
The five worlds. Summary of how we investigate the five worlds in this study.

An important goal of affective computing is to mirror humans’ perception of emotions – in the words of R. Picard, developing machines that *“recognize human emotion, ideally at the same level that people can”* [1, p. 56]. Yet, this is difficult to evaluate due to the intrinsic problems of emotion processing, such as the inherent subjectivity of emotions or the unrealistically restricted number of emotion classes typically used in Speech Emotion Recognition (SER). We will discuss the limitations of (most of) the present-day state-of-the-art approaches towards SER with the metaphor of the ‘five worlds’. We want to go beyond four of these worlds; the first of them we have to live with (cf. Fig 1).

**(i) The fuzzy world**: In automatic speech recognition (ASR), we can assume a sort of ground truth: We can transfer acoustics onto written text and reconstruct the blanks words are surrounded with in written text. The acoustic ground truth can be blurred (variants, noise, slurred pronunciation, and alikes) but it can be disambiguated with the help of phonotactics, lexicon, and syntax, implicitly modelled as well within deep learning (DL) approaches. All this does not exist for emotions. We can ask the speakers which emotions they wanted to produce; by that, they assess their own emotions post festum, and this distorts the ‘reference’ in similar ways as any assessment by others—even by experts—does. There is no clear emotional denotation—emotions per se are constituted by connotations and can be mixed and/or weak. Thus, any gold standard cannot be determined unequivocally—let alone a ground truth. Furthermore, the very nature of emotion is multi-modal, and within SER, it is well established that arousal can be assessed within acoustics; in contrast, valence is predominantly constituted by linguistics/semantics, e. g., within sentiment analysis [2]. In SER, research on emotion categories concentrates on how good they are classified; confusions between categories—in practice, the interpretation of confusion matrices – are not considered in detail. Yet, they are highly relevant—in both human-human and human-machine interaction, there can be fatal confusions and irrelevant ones.
  **Within the fuzzy world**: We cannot overcome the intrinsic problem of fuzziness in emotion modelling. The default way of dealing with it is to face this variability by employing ‘real life’, non-prompted/non-acted emotions [3–6] and more sophisticated labelling schemes [7–9]. In this article, we pursue another approach: We stick to carefully designed acted emotion categories; this enables us to systematically extend the scope beyond the restrictions described in the following, keeping the main object—the (intended) emotion categories—constant. Note that we do not assign any ‘higher value’—or even ‘ground truth status’—to acted emotions; they simply serve as reference categories (gold standard) that can be controlled to a higher extent than real-life data. Fuzziness is taken care of by analysing the confusions between these categories. As we want to exclude the influence of linguistics as intervening factor, we will resort to nonsense speech.
**(ii) The closed world**: First studies in SER typically dealt with data collected *in-vitro*—carefully selected emotional categories [10], such as the ‘big six’ [11], produced by actors and recorded in the lab [12]. These were followed by attempts towards addressing more reality, e. g., by considering elicited, spontaneous speech [3, 4], modelling more realistic scenarios [13], or investigating mixed emotions [6]. Still, most of the machine learning (ML) research is typically hampered by a *Closed World Fallacy* [14]: An ML model’s performance obtained with a few classes cannot be generalised to a real-life scenario where more, confounding classes do exist. Differently, in research on human emotion perception, attempts towards investigating a more realistic scenario by the assessment of ‘recognition’ rather than ‘discrimination’ have been carried out [15]. This was possible by considering ‘distractors’, i. e., providing more emotional classes to the user than those included in the actual data [16, 17].
  **Beyond the closed world**: In order to investigate confusion patterns between emotions in a more realistic setup, we include emotional distractors in both the perceptual and ML tasks. As in our pilot study [18], we use a subset of the *GEneva Multimodal Emotion Portrayals* (GEMEP) database [19], i. e., nonsense speech produced in four emotions (anger, fear, sadness, happiness) with different arousal levels [20].
**(iii) The clean world**: Noise pollution hampers speech communication, reduces comprehension, and can lead to misunderstandings [21]; yet, most of the works in SER investigate data collected in the lab [12]. For assessing the impact of background noise on human understanding of paralinguistic information [22–24], as well as for investigating to which extent SER might be impaired by adverse environmental conditions [13, 25], artificial noise has been typically applied to mask speech recorded in the lab. Differently, real-life noise has seldom been employed [26–28].
  **Beyond the clean world**: In order to evaluate how noisy real-life conditions affect humans and ML when identifying emotions in speech, we systematically compare both perception and classification results obtained from clean and noisified speech. In contrast to our pilot study [18] that employed only artificial noise (brown, pink, and white), we now assess three real-life noises (bell, rain, and train station). From now on, we will refer to the experiments based on artificial noises as EXP-1 and to those based on real-life noises as EXP-2. As in the pilot study [18], the speech was masked with four Signal-to-Noise Ratios (SNRs): -1 dB, -0.5 dB, +1 dB, +3 dB.
**(iv) The small world**: Due to the problems of collecting data and especially annotations [29], emotion corpora are typically small—far away from the size of corpora used for ASR. This can impair the performance of DL systems, which are known to be influenced by the amount of training data. Since DL has revolutionised computer vision [30], there is much interest in other fields on comparatively evaluating whether state-of-the-art ML methods outperform traditional ones [31, 32], and how much performance is impacted by the size of the databases [33]. Yet, this kind of assessment is still rare in the context of SER [34].
  **Beyond the small world**: In order to evaluate to which extent the performance of traditional methods in SER, e. g., of hand-engineered features, is comparable to the one achieved by representations learnt with state-of-the-art DL procedures, we systematically compare the performance of the INTERSPEECH 2013 *Com*putational *Par*alinguistics Challeng*E* (ComParE) feature set [35] with the one of wav2vec2 [36]. For our experiments, we consider standard hyperparameter optimisation, training samples with varying sizes, and two simple ML models: support vector machine (SVM) and multilayer perceptron (MLP). It was necessary to use a small number of items in the initial dataset in order to allow for the comparison ‘human vs machine’; despite the promising results achieved by DL models in SER [37], we thus refrain on their use in the present study, as they require a much larger dataset. (Of note we want to mention that in preliminary experiments, we employed modern DL methods that, however, yielded unsystematic and low performance due to sparse data.) Moreover, as we are interested in investigating a variety of SNRs and types of noises, considering a large initial set of clean samples would make the human annotation task far too costly.
**(v) The one world**: Since the categories ML tries to model are typically based on perceptual human assessment, there is an intrinsic connection between human perception and its encoding in ML. In spite of the rare attempts aimed to comparatively investigate human perception and ML classification [38–41], to the best of our knowledge, a one-to-one assessment guaranteeing identical setups (‘other things being equal’) has never been performed so far. In [42], a first attempt of comparing human perception (previously evaluated in [43]) and ML accuracy is presented. However, neither noise nor distractors, needed to guarantee ‘realistic’ conditions, have been taken into consideration in these two studies.
  **Beyond the one world**: In order to perform a one-to-one comparison between human and machine, we guarantee the same ‘realistic’ conditions by employing noises and distractors for both perception and classification and systematically compare their performances.

**Fig 2:**
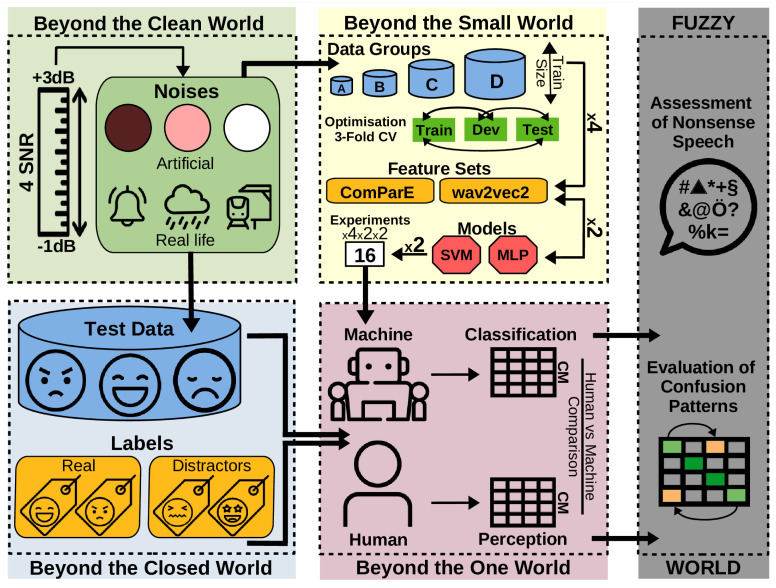
Overview of the study. The methods considered to go beyond the state-of-the-art in the investigated worlds are illustrated: beyond the Closed World (bottom left), both real and distractor labels are used; beyond the Clean World (upper left), 6 types of noise at 4 SNRs are applied; beyond the Small World (upper middle), four data groups with different training sizes, two feature sets and two models are optimised through 3-fold speaker independent cross validation (CV) in 16 experiments; beyond the One World (bottom middle), classification and perception results by machines and humans are assessed through a one-to-one comparison of the Confusion Matrices (CM); in the Fuzzy World (right), the confusion patterns of the perception and classification experiments are evaluated.

Summing up our motivation and the approaches chosen, this study aims to encourage a more adequate modelling and classification of emotions encoded in speech, which is achieved by investigating four specific fallacies beyond the state-of-the-art: First, unlike previous works in SER, which normally concentrate on few emotions while disregarding all other ones, we assess the performance of humans and ML in a more realistic setting, i. e., by assessing their efficiency in handling confounding factors. For that, we introduce so-called ‘distractors’, i. e., emotion classes that have not been seen in the training phase. Second, unlike traditional research, which normally concentrates on clean data, we assess the impact of real-life noise pollution in humans and ML systems while guaranteeing comparable conditions. Third, although ML approaches need a large amount of data, in SER research typically only small datasets are available; we therefore systematically assess how ML performance in SER is impacted by comparing different models, features, and database sizes. Fourth, unlike previous works, where human perception and ML classification are not compared on a strict basis, we perform a one-to-one comparison that enables us to assess the efficiency of ML in emulating human’s perception in SER. In Fig 2, an overview of the different constellations taken into account to systematically assess the described worlds is depicted. Methodological details on each of them are given in the Section *Materials and methods*.

## Materials and methods

### Data and set-up: Beyond the closed world

Given the lack of agreement on the adequacy of the two main emotion models [17], we considered both: the categorical [11] and the dimensional [20]. From the categorical model (with unique discrete classes [11, 44]), we chose the four basic emotions anger, fear, sadness, and happiness. From the dimensional model that represents emotions within a multi-dimensional space [20], we considered high and low levels of the arousal dimension, i. e., intensity. Thus, each of the four categories is encoded in both high and low arousal: *hot anger* and *irritation*, *panicked fear* and *worried fear*, *desperate sadness* and *depressed sadness*, *elated happiness* and *pleasured happiness*. Evaluating these four basic emotions with different arousal levels, referred to as the *four emotional families* [45], is a well-established procedure that enables to assess how a unique category varies over the arousal dimension. It allows to identify confusion patterns between instances with similar quality but different intensity [45]. In addition to the four emotion families, *disgust* and *surprise* were also considered but without arousal connotations. This was decided in order to simplify the set-up as these two emotions, unlike the previous four, are ambiguous concerning their ‘primary/secondary’ status: They are identified as basic by some authors [11] but not by others [46].

**Fig 3:**
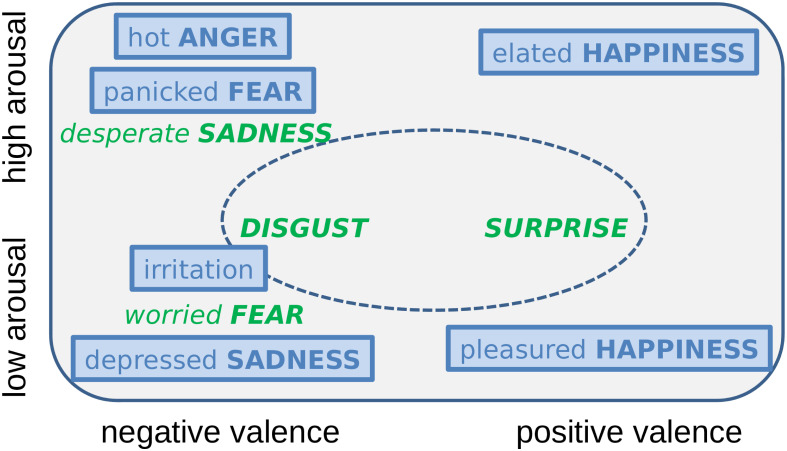
Emotions used in the perception and ML experiments. From the 10 emotions: 6 are ‘real’, whose audio files were used in all perceptual and ML experiments (framed and blue), 4 are ‘distractors’, whose audio files were used only to train the ML models (italics and green); ‘basic’ emotions are capitalised; the inner ellipse indicates no arousal connotations.

To reduce the probability of performing a discrimination rather than a recognition task [15], some emotions were ‘real’, others were ‘distractors’ [16]. ‘Real’ are those represented by audio samples in the listening test and in the ML test set. Distractors are labels for emotion classes provided to our participants that do not correspond to any of the audio samples to be annotated. In the ML task, audio samples of the distractors, i. e., spoken utterances produced by the actors expressing the emotions taken as distractors, were used to train the models but not for test, by that creating similar conditions for the perception and the ML experiment. From the ten emotions, six are real: *hot anger*, *irritation*, *panicked fear*, *depressed sadness*, *elated happiness*, and *pleasured happiness*; four are distractors: *worried fear*, *desperate sadness*, *surprise*, and *disgust*. The procedure how to choose real emotions and distractors is described in more detail in [18]. In Fig 3, real emotions and distractors are displayed.

Previous research has highlighted that the linguistic component of emotional speech affects human perception depending on the listener’s mother tongue [47]: native speakers are much more precise than non-native ones since they use both verbal and non-verbal information when identifying emotions [47]. One strategy to deal with this problem is to take nonsense utterances into account, which prevent any linguistic influence in the listener [48]. In addition, using a standard sentence (i. e., producing the same sentence to express all the evaluated emotions [49]), enables to comparatively assess how emotions are identified, based on their acoustic characteristics, while keeping the verbal component and by that, the phonetic/phonological content, stable.

Thus, to avoid a linguistic bias, i. e., the influence of linguistic meaning on the listeners, the nonsense utterance *Ne kal ibam soud molen!* from the GEMEP database [19] was used. The nonsense utterance consists of a pseudo-linguistic phone sequence based on phonemes as they can be found in several Western languages; thus they give the impression of a real utterance produced in a foreign language [24]. Note that a nonsense utterance, by definition, does not have any meaning in any language; thus, it is not expected to be understood by the reader. The utterance was produced by six French actors (3 female, 3 male). In total, 36 instances from GEMEP are considered: 1 utterance x 6 speakers x 6 emotions (duration *μ* = 2.57 sec., *σ* = 0.77 sec.). As the phonetics of a nonsense utterance might resemble a specific language, thereby influencing the emotional understanding of a native of such a language [48, 50, 51], we recruited a homogeneous group of Italian listeners: 132 engineering students from *Tor Vergata* university (55 female, 77 male; age *μ* = 20.7 years, *σ* = 2.5 years). The perception experiment was hosted on a browser-based interface provided through the gamified crowd-sourcing platform *iHEARu-PLAY* [52], and presented over headphones as a forced-choice task. The stimuli were randomised differently for each participant; they could select only one out of the 10 emotions. Informed consent was obtained through the platform; the volunteering participants obtained credits but remained anonymous and provided only gender and age. Since the research processes carried out cannot affect the physical or psychological integrity of the study participants, the Ethics Committee of the University of Augsburg confirmed that ethical approval was not necessary for this study.

### Real-life noise: Beyond the clean world

**Fig 4:**
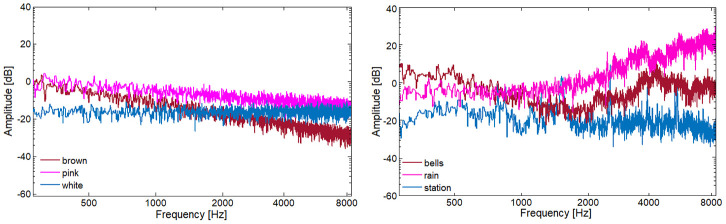
Spectral distribution. Frequencies between 0–8 kHz (most important for speech) and amplitudes between -40 to 40 dB, are shown for the artificial (brown, pink, white) and the real-life (bell, rain, train station) noises. All samples have 10 sec. length (Root Mean Square is normalised).

To mask the emotional speech, three real-life noises from different soundscapes [26] were chosen from the web-dataset freesound: bell (rural), rain (nature), and train station (urban); cf. Fig 4 (right). Each noise (10 sec. length) was mixed with the speech items at four Signal-to-Noise Ratios (SNRs): -1 dB, -0.5 dB, +1 dB, and +3 dB. In [18], these SNRs have produced clear and systematic differences in the perception of noisified emotional speech. Since utterance length varies across speakers and emotions, the noise segments used to mask every instance were randomly selected. Although from real-life, the chosen noises are homogeneous, by this guaranteeing a comparable masking across samples. The noises can be freely downloaded at: https://drive.google.com/drive/folders/1nxF2EbRcYVJp9ce5OwqIUsrMr1AdvL5O.

The artificial noises used in [18] (brown, pink, and white) are displayed in Fig 4 (left). The bell noise, up to approx. 1.5 kHz, presents a trend similar to brown noise, with a fall of energy around 6 dB per each doubling in frequency; above 1.5 kHz, this trend is inverted, increasing around 6 dB per each doubling in frequency. The rain noise has a steady power density up to 1 kHz and an increment of around 3 dB per each doubling in frequency above 1 kHz—thus, an inverse trend w. r. t. pink noise. The train station noise, showing an almost equal distribution of energy across all the frequency bands, is similar to white noise. Note that here, we compare only the magnitude spectra of our signals and disregard other, more fine-grained characteristics. In total, 432 ‘noisified’ stimuli were generated (36 instances x 3 noises x 4 SNRs), resulting in 468 stimuli (432 noisified + 36 clean). Due to the big amount of stimuli, to avoid fatigue, they were randomly assigned to four sessions (each of 45 min.).

Since the real-life noises were applied with a specific noise type and SNR, our samples might not perfectly reflect real environments, which vary over time in quality and intensity. Thus, to assess the validity of the audio samples, the listeners rated also whether the noisified instances were produced in a real-life situation. In more than 75% of the cases, with no marked differences between the highest (+3 dB) and the lowest (-1 dB) SNR, Pearson’s chi-squared yielded *p* ≥.175 for all the comparisons, i. e., listeners perceived the noisified samples as produced in real-life conditions. This is not surprising, considering the short length of the instances (*μ* = 2.57sec., *σ* = 0.77sec.), for which we might assume a sort of steadiness also for in-the-wild samples. In real-life situations, people might increase their vocal effort when exposed to noise; this *Lombard effect* is characterised by an increment in amplitude, pitch, and spectral variations [53]. As each emotion has typical acoustic traits [45], which differ between Lombard and non-Lombard speech, findings from Lombard speech would hardly be comparable to previous works that mostly evaluate non-Lombard speech. Moreover, speech need not necessarily be altered in noisy environment, when, e. g., two dialogue partners are close together; yet, it will be more difficult for listeners further away to understand their conversation.

### Machine learning: Beyond the small world

**Fig 5:**
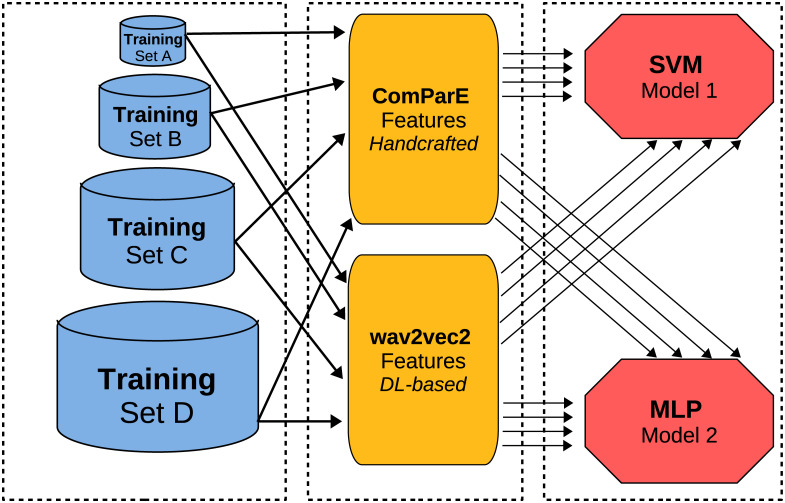
Experimental design. Main components of the ML workflow: Data groups (A, B, C, and D) represented according to the diverse sizes of their training set; Feature sets (ComParE and wav2vec2); and ML models (SVM and MLP).

The diagram in Fig 5 illustrates the workflow of the ML implementation designed to assess how different sizes of the training set as well as features and architectures impact a model’s performance in an SER task. The model training and evaluation are performed for 4 differently-sized training sets (corresponding to the four data groups A, B, C, and D), considering 2 independent feature sets, and 2 ML approaches, resulting in 16 experiments.

#### Features and models

We evaluate the performance of two ML models: (i) an SVM classifier; although this is considered to be overtaken by more sophisticated approaches, mainly Deep Neural Networks (DNNs), it is still competitive in SER [35, 54]; (ii) an MLP, i. e., a classical fully-connected feed-forward neural network.

The models were fed with traditional hand-engineered features (ComParE) and with state-of-the-art DL-based embeddings (wav2vec2). ComParE [35] is a feature set tailored for SER which encompasses 6 373 acoustic features divided into four sub-sets: Mel-Frequency Cepstral Coefficients (MFCCs), spectral features, prosodic features, and voice quality features. They are computed by applying statistical functionals, including extremes, percentiles, moments, and linear predictive coding coefficients, to 65 Low-Level Descriptors (LLDs) and their delta coefficients. ComParE features are extracted using the default parameters of the openSMILE toolkit [55], i. e., a Hamming window of 20 ms for the MFCCs and spectral features, and a Gaussian window of 60 ms for the prosodic and voice quality features; all the LLDs were extracted with a 10 ms hop size. The models were fed with the functionals, i. e., each instance was represented as a vector of length = 6373.

In addition, features are extracted using the wav2vec2 model, a deep neural network, operating on the raw waveform and consisting of convolutional and *transformer* [56] layers. The network is typically first pre-trained on large amounts of speech data in a self-supervised way, i. e., predicting embeddings of randomly masked timesteps in each audio sequence, and then fine-tuned on a target task. In this work, the model published by Wagner et al. [57] is employed, which has been pre-trained on four large speech corpora and then fine-tuned on emotion recognition in terms of arousal, valence, and dominance, using the MSP-Podcast corpus [58]. As features, we use the outputs of the last transformer layer, with an average pooling across all frames of each audio file. With this approach, each instance was represented as a vector of length = 1 024.

#### Data groups

**Fig 6:**
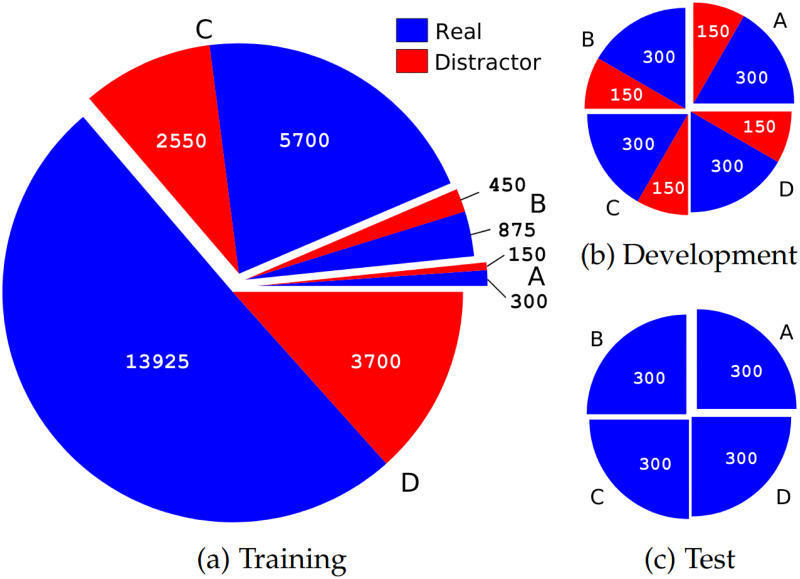
Distribution of real samples and distractors. Partitioning across the three sets (training, development, test) and data group (A, B, C, D) is indicated. The distribution of speakers is: Training (A = 2, B and C = 6 each, D = 16; Development and Test (A, B, C, D = 2 each).

Since DL approaches are said to be more successful with higher numbers of samples, the experiments were performed on four data groups (A, B, C, and D) varying in the size of the training set. The four data groups encompass the same emotions: 6 real (considered in all partitions), 4 distractors (considered only for ML training and optimisation, i. e., the tuning of the models’ hyper-parameters described in the Section *Model Optimisation*). In Fig 6, the distribution of samples across data groups and partitions is given (cf. Section *Partitioning* for further details).

**(i) Data group A**: 1 350 instances (900 real + 450 distractors). The 900 real are those from the two perceptual studies (EXP-1 and EXP-2): 36 clean (6 speakers x 1 utterance x 6 emotions) + 864 noisified (36 clean x 6 noises x 4 SNRs). The 450 distractors are produced by the same 6 speakers on the same utterance: 18 clean (6 speakers x 1 utterance x 4 emotions) + 432 noisified (18 clean x 6 noises x 4 SNRs). Note that 6 out of the 24 clean expected instances were missing from GEMEP (3 for surprise, 3 for disgust), thus only 18 were considered.
**(ii) Data group B**: 2 225 instances (1 350 from A + 875 new). The 875 new (575 real, 300 distractors) are produced by 4 additional speakers (2 female, 2 male) on the same utterance and emotions used in A: 35 clean (4 speakers x 1 utterance x 10 emotions) + 840 noisified (35 clean x 6 noises x 4 SNRs). Note that 5 out of the 40 clean expected instances were missing from GEMEP (2 for disgust, 2 for surprise, 1 for cold anger), thus, only 35 were considered.
**(iii) Data group C**: 9 150 instances (2 225 from B + 6 925 new). The 6 925 new (4 825 real, 2 100 distractors) are produced by the 4 additional speakers from B, on the nonsense utterance *Koun se mina lod belam?* and the sustained vowel *[a:]* (used to increase the amount of instances) in the 10 emotions: 277 clean (unbalanced across speakers and emotions in GEMEP) + 6 648 noisified (277 samples x 6 noises x 4 SNRs).
**(iv) Data group D**: 18 525 instances (9 150 from C + 9 375 new). The 9 375 new (8 225 real, 1 150 distractors) are instances from emoDB [12], produced by 10 German actors (5 female, 5 male) on a variety of utterances and 5 emotions: 375 clean + 9 000 noisified (375 samples x 6 noises x 4 SNRs). From the 5 emotions, 4 were real (hot anger, panicked fear, depressed sadness, and elated happiness), one was a distractor (disgust). Note that the sentences in emoDB are neutral, i. e., void of any ‘emotional connotation’, which makes them comparable to the utterances from GEMEP. emoDB is not used in the perception experiments; thus, non-nativeness does not play any role.

#### Partitioning

In the following, we introduce the data partitioning across experiments and how this relates to the ML optimisation. In ML, an *experiment* is a (classification) task conducted with a specific feature set, model, partitioning, and data group. *Partitioning* aims at distributing the data points onto the three (speaker-independent) sets: training (used to train the model); development (used to optimise the model’s hyperparameters); and test (used to test the model’s performance).

To perform a subject-independent task, the samples produced by a pair of speakers (1 female, 1 male) out of the 6 speakers evaluated in the perceptual study was kept for the test set, while the remaining 4 were considered for training and development; additional speakers are used for training in groups B, C, and D. To prevent a speaker-related bias, the experiments were carried out three times, by considering each time a different pair in the test set, i. e., three permutations of the pairs. Subsequently, the results across the three experiments are averaged. For comparability, the same pairs were used for the three test sets in all the experiments regardless of the data group; each test set was made up of 300 samples: 12 clean (2 speakers x 6 real emotions), 288 noisified (12 clean samples x 6 noises x 4 SNRs). In Fig 6, the distribution of samples is given. Note that the sum of distractors per data group (A, B, C, D) indicated in Fig 6 is lower by 150 than the one given in the description of the data groups. This is because no distractors are considered in the test set, cf. Fig 6, although they do exist in every data group, since needed to perform the permutations across sets.

For optimisation, a three-fold nested Cross-Validation (CV) was chosen. Evaluations were carried out individually for each acoustic condition (clean and noisy ones) and SNR level, as well as combining all the samples together. In order to guarantee a fair comparison with the perception results, for the classification of individual conditions, the training was, however, carried out on all the conditions. Although this makes the ML task more challenging, it allows a one-to-one comparison since the previous knowledge from humans can be considered, to some extent, comparable to the knowledge of a model trained on multiple conditions. In addition, for the MLP, early stopping was applied to avoid over-fitting.

#### Model optimisation

To optimise the models, we considered a reduced range of values for specific hyper-parameters. We do not concentrate on pushing one specific approach towards its limits but on comparing the approaches based on ‘standard’ settings, which could be employed in a more generic scenario, i. e., beyond a specific dataset. To make a fair comparison between humans and ML, the models were trained and optimised on the recognition of the 10 emotional classes, i. e., real and distractors were considered in the training and development sets. By this, we infer in the models a knowledge about the emotional classes used as distractor similar to the one that a human (exposed to these emotion) would have. Differently, as performed in the perceptual study, the test set contained only samples of the real classes. Note that our goal is not to achieve the best possible performance through optimisation but to understand how traditional and state-of-the-art methods perform in comparable settings.

**(i) Support Vector Machine**: We used an SVM with linear kernel built on the scikit-learn python library [59]. For its optimisation, we tuned the complexity (C): 5 different C on a logarithmic scale (from.00001 to.1) were evaluated. Subsequently, the SVM was trained again (considering the training and development sets together as a unique set) with the C which yielded the highest Unweighted Average Recall (UAR, i. e., the arithmetic mean of the recalls of the 10 classes) during optimisation. Due to the unbalanced distractors, UAR for the development set was considered.
**(ii) Multilayer Perceptron**: We built an MLP on TensorFlow 2.6 [60] through the API Keras [61] and optimised it according to three hyper-parameters: Number (N) of hidden layers and neurons, learning rates, and batch sizes; for each, we evaluated three different values. The N of hidden layers and neurons was set out considering for each deeper hidden layer (from 1 to 3) a decrease by half in the N of neurons (from N neurons = N features/2 in layer 1 to N features/8 in layer 3). The values for the learning rate were:.01,.001,.0001; for the batch size: 50, 100, 200. Beyond evaluating all the combinations of these values, other hyper-parameters were set out: Adam optimiser, Rectified Linear unit (ReLu) activation functions in the hidden layers, Softmax activation function in the output layer, a maximum of 100 epochs, a dropout of 20%, and early stopping with a patience of 10.

To enhance the robustness of the results, each experiment is repeated 5 times using a different random initialisation of the model in each iteration.

### Human vs machine: Beyond one world

To the best of our knowledge, one-to-one comparisons between human perception and state-of-the-art ML models based on identical settings have not been performed so far. As for a more traditional approach, see [38], where a linear classifier has been employed. For a comparison between perception and ML within a cross-lingual setup, see [40, 62]. For Japanese, see [42, 43]. However, these studies do not use distractors; thus, to which extent emotional confusion patterns displayed by listeners might be mirrored by state-of-the-art ML is not clear yet.

To enable a fair comparison between human and ML, instances expressing emotional distractors were used for training and optimising the ML models, but not for testing them. This makes the two tasks comparable since the distractors are learnt by the system, although they are no test targets. To avoid complicating the ML experiments carried out individually on each noise, all the SNRs were considered together; when performing the one-to-one comparison, the perceptual results obtained from the four different SNRs were taken together as well. For the perceptual and ML comparison (cf. Section *Human vs machine: Assessing the two worlds*), we use the best performing model, by this aiming to illustrate to which extent ML methods emulate (or at least mirror) human perception of emotion. Although special efforts were taken to make the tasks comparable, we might still consider the task being slightly harder for humans, as they have background knowledge but no specific training.

## Results

We report Unweighted Average Recall (UAR) for all experimental results, i. e., the mean of the class-wise recall in percent from the diagonal in the confusion matrices. Here, the UAR is equal to the weighted average recall as in the perceptual study and the test set for the ML task, the class frequencies are fully balanced. We also discuss *recall* (true positives divided by the total number of samples per class) and *precision* (true positives divided by the total of ‘recognised as’ per class). Beside the standard evaluation metrics UAR, recall, and precision, we report also sums of *‘identified as’* per class; this is meaningful for interpreting the confusion patterns towards specific emotions.

Due to the intrinsic problems of Null Hypothesis Testing [14, 63], throughout this article—if appropriate—we display *p*-values from two-tailed Pearson’s chi-squared with Bonferroni adjustment as descriptive measures, not as criteria deciding between hypotheses. By that, we provide the traditional measure for readers expecting p-values—however, without employing this paradigm ourselves.

### Perceptual study

Confirming the outcomes of the pilot study [18] on artificial noise (EXP-1), the results from EXP-2 employing real-life noise show that the higher the noise intensity, the lower is the UAR. Since this holds for all noise types, from now on only results for the most affecting SNR (-1dB) will be discussed here; for results across all SNRs, see Section *Human vs machine: Assessing the two worlds*.

#### Role of distractors: Assessing a more open world

Perceiving female voices, female listeners achieved a mean UAR of 37.8% across conditions, male 34.1%. Perceiving male voices, female listeners achieved 27.8%, male 24.1%. Due to these small differences (*p* = .116 for female voices, *p* = .085 for male ones), from now on, both listeners’ genders will be evaluated together. Similarly, none of the differences on the perception of female and male voices was marked: Across conditions, females are perceived with a mean UAR of 34.4%, males with 24.6% (the smallest *p*-value was *p* = .163). Hence, listeners’ responses will be evaluated disregarding speakers’ gender as well.

As the recognition of the reference categories is more challenging than their discrimination, it could be expected that the distractor labels stimulated the spread of the responses, shown by a low Fleiss’ kappa inter-rater agreement (*k* = 0.22 in EXP-1, *k* = 0.20 in EXP-2). However, the distractors yielded values lower than 25% for all emotions except worried fear, where they amount to 28.1% in the most noisy environment, i. e., rain noise at -1 dB. Confirming previous findings [45, 64, 65], depressed sadness (from now on referred to as ‘sadness’), was the emotion best recognised: mean recall of 61.9% in EXP-1 and 62.5% in EXP-2 (cf. Table 1); *p* <.001 in all the comparisons except sadness vs irritation in EXP-1 (*p* = .169).

**Table 1:** Perceptual results for clean and noisified conditions.

%		Hot Anger	Irritation	Sadness	Panic Fear	Elation	Pleasure	UAR
EXP-1	clean	**59.5**	**64.3**	**58.6**	29.8	30.1	28.1	45.1
	brown	34.1	**61.3**	**66.2**	21.7	18.5	18.9	36.8
	pink	23.1	40.1	**59.0**	19.3	11.2	15.1	28.0
	white	31.5	**51.0**	**63.6**	20.0	17.3	12.6	32.7
mean		37.1	**54.2**	**61.9**	22.7	19.3	18.7	35.7
EXP-2	clean	43.5	**50.8**	**61.3**	31.0	29.7	13.8	38.3
	bell	18.9	41.0	**61.8**	21.4	20.6	5.1	27.9
	rain	14.4	29.4	**65.7**	15.8	18.6	6.1	25.0
	station	14.2	37.9	**61.1**	16.9	21.8	6.7	26.5
mean		22.7	39.8	**62.5**	21.3	22.7	7.2	29.4

Noise at -1 dB SNR: artificial (EXP-1), and (EXP-2). For each condition, 36 samples (6 per emotional class), assessed by 26 users in EXP-1 and 132 users in EXP-2. Results for female and male listeners are aggregated. Recall per class, UAR, and mean across conditions for recall and UAR are given (values above 50% in bold).

Sadness was best recognised due to the fact that all emotions are perceived to some extent attenuated in background noise, as already proved in [18] by varying the SNR level. This ‘attenuation’ gives the impression of lower energy and pitch [66] to the other emotions, corresponding to acoustic characteristics typical for sadness and to some extent for irritation. By that, this creates a strong confusion towards these two low aroused emotions, particularly for sadness, which due to this bias shows a higher recall in background noise than in clean condition. In order to identify the direction of the main confusion patterns among emotional categories, i. e., to understand which emotions attract more confusion, we evaluate the sums of responses ‘identified as’, i. e., the ‘correct’ (hits) + the ‘incorrect’ (false alarms) given for each emotion (cf. Table 2). With hits we refer to the number of ‘correct’ responses, i. e., the samples from each emotional category correctly perceived by the listener; with false alarms we refer to the number of ‘incorrect’ responses, i. e., the samples misclassified with a given emotional category although expressing another emotion. The emotion mostly chosen was sadness, with a mean across conditions of 142.1% for EXP-1 and 162.8% for EXP-2. Second, as expected, comes irritation: mean of 133.6% for EXP-1, 125.3 for EXP-2. All the other emotions were below 100% for ‘identified as’.

**Table 2:** Sums of ‘perceived as’ (hits and false alarms).

%		Hot Anger	Irritation	Sadness	Panic Fear	Elation	Pleasure	mean
EXP-1	clean	78.0	**124.6**	**102.6**	86.0	34.2	83.8	84.9
	brown	46.5	**147.4**	**139.0**	77.9	29.2	78.6	86.4
	pink	37.2	**135.6**	**168.9**	75.7	23.8	68.1	84.9
	white	49.3	**126.9**	**157.8**	80.9	27.1	65.0	84.5
mean		52.8	**133.6**	**142.1**	80.1	28.6	73.9	85.2
EXP-2	clean	61.4	**131.3**	**130.0**	65.5	43.2	45.5	79.5
	bell	31.3	**127.3**	**163.4**	61.1	40.1	54.6	79.6
	rain	23.6	**118.5**	**186.2**	50.9	35.2	51.9	77.7
	station	26.2	**125.7**	**171.7**	53.9	38.5	51.4	77.9
mean		35.6	**125.3**	**162.8**	57.9	39.3	50.9	78.6

Results for clean and noisified conditions (at -1 dB SNR) are given. For each, 36 sample (6 per emotional class), assessed by 26 users in EXP-1 and 132 users in EXP-2. Results for both female and male listeners are aggregated. Means per emotion across conditions, and per condition across emotions are given (values above 100% in bold).

#### Clean vs noise: Assessing a noisy world

As expected, the clean samples are those recognised best in both experiments: 45.1% in EXP-1, 38.3% in EXP-2; cf. UAR for clean in Table 1. The three real-life noises affected the listeners similarly: Samples noisified with bell noise are perceived slightly better (27.9%), those with rain noise slightly worse (25.0%), those with train station noise in between (26.5%); cf. UAR for EXP-2 in Table 1. With higher differences, this is also observed in EXP-1: brown noise affected less (36.8%), pink noise most (28.0%), white noise in between (32.7%); cf. UAR for EXP-1 in Table 1. These trends can be interpreted, to some extent, according to the acoustic characteristics of the noises: bell similar to brown, rain to pink, train station to white. Yet, all these differences are minimal (*p* ≥.348).

**Fig 7:**
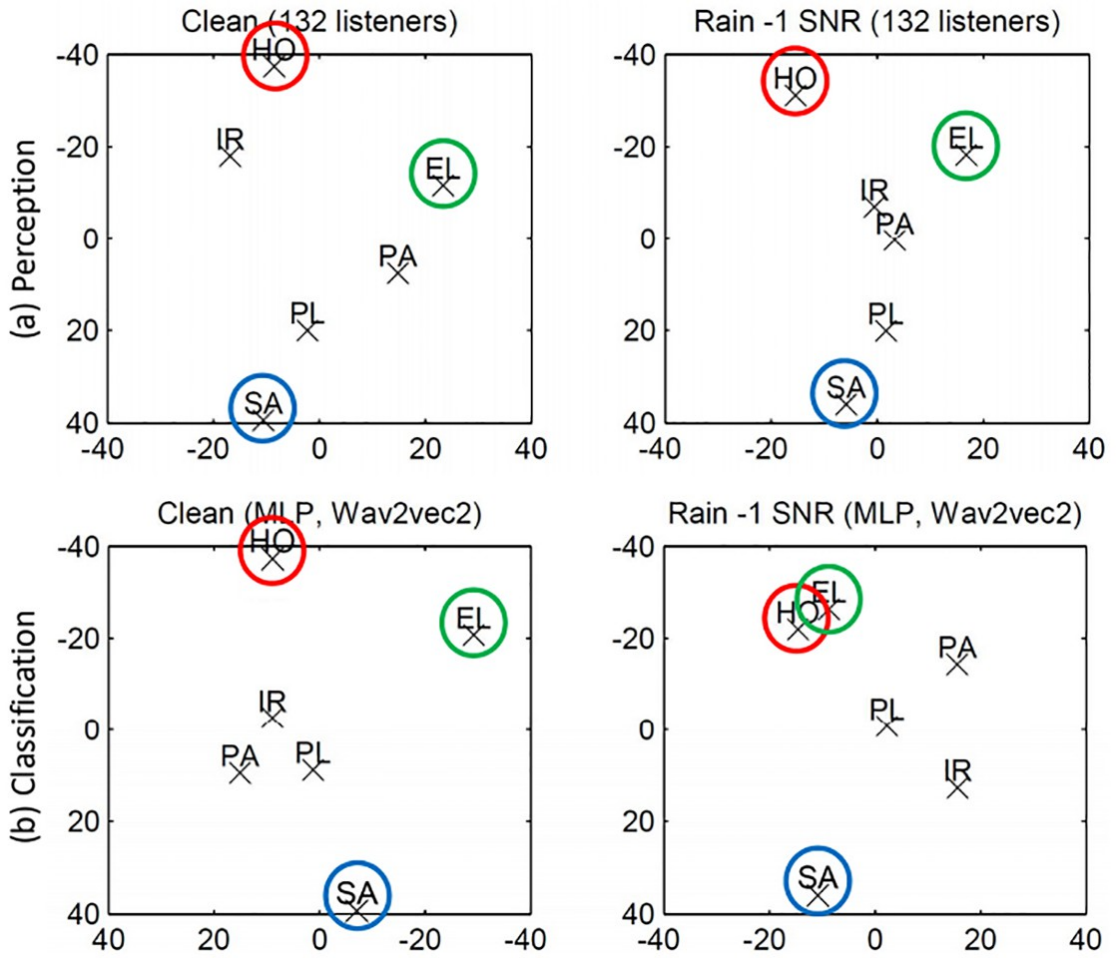
Non-Metric Multi-Dimensional Scaling (NMDS). The 2-dim(ensional) solutions represent (a) listeners’ perception and (b) automatic classification: hot anger (HO), panicked fear (PA), irritation (IR), depressed sadness (SA), elation (EL), and pleasure (PL); in clean and in rain noise. Kruskal’s stress for perception in (a): Clean (.115); Rain noise (.036); for classification in (b): Clean (.150); Rain noise (.114); bottom left, the x-axis is mirrored to display the dimensions similarly for perception and classification.

In order to visualise perception in a sort of ‘cognitive space’, 2-dim(ensional) *Non-Metric Multi-Dimensional Scaling* (NMDS, [67]) solutions for the confusion matrices for clean and the most disturbing real-life background (rain noise at -1dB), displayed in Table 3, are given in Fig 7(a). These results are comparable to the NMDS solutions presented in [18] for EXP-1, for clean and pink noise at -1 dB; thus, only the results of EXP-2 will be discussed. The NMDS represents the non-metric optimal distances between the emotion categories. Starting with a random configuration of points, the NMDS tries to find the optimal proximity between points, i. e., the interpoint distances configuration, taking into account the dissimilarities between the classes [68]. The stress value between the optimally scaled data (in a reduced dimensionality) and the distances are optimised by finding a new configuration of points. This is iterated until a criterion is met. NMDS is one amongst several graphical representations highly useful for Exploratory Data Analysis (EDA) and visualising constellations that are difficult to see in the confusion matrices the NMDS is based on.

**Table 3:** Confusion matrix for the perception of emotions by 132 listeners.

%		Hot Anger	Irritation	Sadness	Panic Fear	Elation	Pleasure	distractors
clean	Hot Anger	43.5	35.7	2.3	4.3	2.3	2.8	9.2
	Irritation	7.3	**50.8**	12.3	2.4	3.3	7.0	17.0
	Sadness	1.1	11.0	**61.3**	1.0	2.5	4.1	18.9
	Panic	2.2	10.9	17.9	31.0	2.3	3.0	32.7
	Elation	6.1	13.1	3.2	10.7	29.7	14.9	22.3
	Pleasure	1.3	8.2	33.1	16.1	3.2	13.8	24.3
precision		**70.7**	39.1	47.2	47.3	**68.6**	30.3	–
rain SNR -1	Hot Anger	14.4	40.8	7.0	7.7	4.1	6.7	19.3
	Irritation	2.6	29.4	23.7	2.9	3.8	12.1	25.6
	Sadness	1.1	10.3	**65.7**	2.7	2.4	3.8	13.9
	Panic	2.4	14.5	29.5	15.8	3.2	6.5	28.1
	Elation	1.7	14.4	11.8	12.0	18.6	16.8	24.7
	Pleasure	1.4	8.9	48.4	9.8	3.0	6.1	22.4
precision		**61.0**	24.8	35.3	31.1	**52.9**	11.7	–

For each emotion, 6 instances (3 produced by females, 3 by males) were assessed in clean and rain noise at -1 dB SNR. Precision and cases misclassified as distractors are indicated. For simplicity, and since distractors are not represented in the NMDS in Fig 7(a), percentage of cases misclassified as any of the distractors are given together. Lower values for the distractors are associated with a higher perception accuracy and vice-versa. Darker shadowing indicates higher percentage, values >50% are boldface. The confusion matrices are basis for the NMDS in Fig 7(a).

In Table 3, the confusion matrices, the NMDSs shown in Fig 7(a) are based on, are given; note that Fig 7(b) will be discussed further below. Sadness, irritation, and hot anger, perceived with a recall of 61.3%, 50.8%, and 43.5%, respectively, are the emotions best recognised in clean condition; cf. the diagonal in Table 3. Due to the confusion attracted by sadness in adverse environmental conditions, this still holds in background noise: 65.7%; cf. sadness for Rain in Table 3. No marked difference is shown for the recognition of sadness between the noisy and the clean condition (*p* = .076). This can also be seen in the NMDS, where sadness is represented at an extreme position in both clean and noisy conditions; cf. SA in Fig 7(a). Although irritation and hot anger are recognised worse in noisy than in clean condition (*p* <.0001), they are also represented at more extreme positions—across from sadness—in both clean and noisy background; cf. IR and HO in Fig 7(a). This is most evident for hot anger and indicates that its confusion with sadness is always minimal regardless of the condition, i. e., they are perceived as clearly different from each other. The percentage of utterances expressing sadness misclassified as hot anger is the same in clean and background noise: two times 1.1%; see Table 3.

Pleasure is the emotion worst recognised, with the lowest recall and precision in both conditions: 13.8% and 30.3% for clean, 6.1% and 11.7% for rain SNR -1, for recall and precision, respectively; cf. Table 3. Yet, no marked differences are shown w. r. t. the next worse recognised emotions, i. e., elated happiness in clean and panic in noisy conditions: *p* = .101 and *p* = .538, respectively. This might indicate that pleasure, followed by panic and elation, has a lower prototypicality [34], i. e., its expression might acoustically not be clearly defined, resulting in confusions with other emotions. Indeed, pleasure and to some extent panic are displayed rather in the central area of the NMDS; cf. PL and PA in Fig 7(a), indicating a lower dissimilarity between them.

### Machine learning approach

Since no marked differences were shown between the perception of male and female voices, the ML experiments were performed considering all the samples together, irrespective of speakers’ gender.

#### Data groups: Assessing a bigger world

In Table 4, the performance of each feature set for each model on the evaluated data groups is given; to focus on evaluating the role of the training set size, noisified and clean samples are considered together. Note that the performance for clean vs noisified is given in Section *Clean vs noise: Assessing a noisy world* and the performance of ML for each different noise individually is given in Section *Human vs machine: Assessing the two worlds*. For both feature sets and models, the UAR generally increases with the size of the training set. This tendency is more pronounced for the wav2vec2 than for the ComParE feature set. SVM trained with ComParE yielded the lowest difference between the smallest and biggest data groups: 20.3 vs 24.3 (*p* = .239); SVM trained with wav2vec2 yielded the highest one: 26.7 vs 35.8 (*p* = .017); cf. UAR for A vs D in Table 4). This shows, as expected, that using a larger training set and state-of-the-art features impacts performance positively.

**Table 4:** ML results considering all conditions together.

**ComParE features**								
%		Hot Anger	Irritation	Sadness	Panic	Elation	Pleasure	UAR
SVM	A	42.0	20.7	34.7	6.0	14.0	4.7	20.3
	B	46.0	22.0	36.7	11.3	12.0	8.0	22.7
	C	32.7	30.0	32.7	20.0	18.7	12.7	24.4
	D	26.0	28.7	32.7	29.3	17.3	12.0	24.3
mean		36.7	25.4	34.2	16.7	15.5	9.4	22.9
MLP	A	33.2	30.3	27.2	11.2	24.1	12.1	23.0
	B	41.1	30.5	27.5	16.3	29.1	7.7	25.4
	C	39.9	36.8	29.3	24.0	31.6	13.3	29.2
	D	46.3	43.5	27.1	31.7	30.3	12.5	31.9
mean		40.1	35.2	27.8	20.8	28.8	11.4	27.6
**wav2vec2 features**								
%		Hot Anger	Irritation	Sadness	Panic	Elation	Pleasure	UAR
SVM	A	50.0	15.3	54.0	4.0	30.7	6.0	26.7
	B	44.7	22.0	52.0	10.0	22.0	10.7	26.9
	C	50.0	34.0	63.3	9.3	12.7	15.3	30.8
	D	55.3	12.7	74.7	32.7	31.3	8.0	35.8
mean		50.0	21.0	61.0	14.0	24.2	10.0	30.1
MLP	A	44.1	13.2	47.3	23.5	28.5	11.7	28.1
	B	42.7	19.1	52.1	27.6	29.5	24.5	32.6
	C	47.2	25.7	57.9	26.4	33.3	13.6	34.0
	D	51.3	27.5	56.0	29.5	29.9	21.1	35.9
mean		46.3	21.4	53.3	26.8	30.3	17.7	32.7

Recall per class, UAR, and mean across data groups (A, B, C, and D) are given for each model (SVM, MLP) with both ComParE and wav2vec2 features. Note that the size of the training set increases across data groups from A (smallest) to D (largest). For more details, cf. subsection *Data groups* and Fig 6.

In order to investigate the distractors’ impact, the experiments were also performed for the data group A without distractors (i. e., also not in the training set). This yielded, as expected, better performance, in particular for SVM with ComParE features, which presents the highest differences between UAR with and without distractors: 20.3% vs 29.6%; cf. SVM for ComParE in Tables 4 and 5 (upper part) for results with and without distractors (*p* = .008). This suggests that hand-engineered features, which do not have the advantage of being computed by an ML model trained with a large amount of data, might be more sensitive to realistic conditions, especially when a simple model such as SVM is used. In contrast, the wav2vec2 features are extracted from a model tuned specifically to recognise emotion in terms of the three continuous dimensions arousal, valence, and dominance [57]. Due to the high amount of training data involved in the generation of wav2vec2 features, these representations should be sensitive to a large variety of emotions, including the distractors; thus, it might be easier to separate the distractors from the 6 real emotions when training the classifier on all 10.

**Table 5:** ML performance excluding the distractors from the training set.

Group A (all conditions)							
	Hot Anger	Irritation	Sadness	Panic	Elation	Pleasure	UAR
**ComParE**							
SVM	43.3	38.0	31.3	22.7	34.7	7.3	29.6
MLP	26.0	33.5	23.9	16.5	39.3	14.8	25.7
**wav2vec2**							
SVM	61.3	14.7	68.0	11.3	43.3	4.0	33.8
MLP	43.9	28.4	46.3	21.5	39.2	21.1	33.4
mean	43.6	28.7	42.4	18.0	39.1	11.8	30.6
Group D (all conditions)							
	Hot Anger	Irritation	Sadness	Panic	Elation	Pleasure	UAR
**ComParE**							
SVM	32.0	36.0	28.7	42.7	28.0	16.7	30.7
MLP	42.9	44.9	30.0	33.9	42.4	14.1	34.7
**wav2vec2**							
SVM	55.3	16.0	67.3	36.7	48.0	17.3	40.1
MLP	53.5	39.7	62.0	30.5	47.2	17.6	41.8
mean	45.9	34.2	47.0	36.0	41.4	16.4	36.8

Results are given for data groups A and D (without distractors, all conditions). Recall per emotion and UAR are given for the performance of SVM and MLP, with ComParE and wav2vec2 feature sets. Mean across models is also indicated.

#### Role of distractors: Assessing a more open world

Hot anger and sadness are generally the emotions best recognised: for wav2vec2, i. e., the best performing feature set, on average 50.0% and 61.0% for SVM; 46.3% and 53.3% for MLP, respectively. Pleasure was worst recognised: on average across data groups ≤17.7% for both models and features; cf. mean recall in Table 4. When evaluating the models in optimised conditions, i. e., without distractors, the same tendency can be observed: across both models and feature sets, hot anger is the emotion best recognised (43.6%), pleasure the worst (11.8%); cf. mean in the upper part of Table 5. To further assess whether this trend persists in optimal conditions, we evaluate data group D with wav2vec2 features that yielded the best ML results, but this time without distractors. Confirming the perceptual results, the ML experiments on data group D without distractors show that sadness is best recognised, pleasure worst: 47.0% and 16.4%, respectively; cf. mean in the lower part of Table 5. This can be explained by the emotions’ level of prototypicality [34]: Sadness, having a more standardised representation, is classified best; pleasure, less standardised, worst. With wav2vec2 features, except for hot anger and elation (*p* >.05), sadness is markedly better recognised than all the other emotions (*p* ≤.003). Concerning UAR, the best results were reached again with the MLP and wav2vec2 features (cf. 41.8% in the lower part of Table 5).

#### Clean vs noise: Assessing a noisy world

To further evaluate the influence of noise on the classification of each emotion, the set-up leading to the best performance with distractors, i. e., the MLP with wav2vec2 features trained on data group D (cf. 35.9% UAR in Table 4), was tested separately for the recognition of clean and noisy samples. In order to enable a fair comparison with human perception, the models already trained with all noise conditions were used. As previously shown for perception, in Table 6, the confusion matrices for the classification of clean data (UAR = 45.0%) and rain noise at -1 dB SNR (UAR = 31.7%), are given. As expected, the classifier performed best without any background noise. Confirming the results from the perceptual study (cf. Table 3), this becomes evident for hot anger, with a decline in recall of more than half between clean and noisy conditions: 66.7% vs 30.0% (cf. clean vs rain for hot anger in Table 6). Similarly as shown by the listeners, the decline in recall for irritation in background noise is due to a more pronounced confusion pattern towards low aroused emotions, i. e., sadness and pleasure: In clean background, 3.3% and 20.0% of irritation samples are misclasified as sadness and pleasure, respectively; in rain noise at -1 dB SNR, the misclassification raised to 20.0% and 30.0%, respectively (cf. clean vs rain for irritation in Table 6).

**Table 6:** Confusion matrix for the classification of data group D with MLP and wav2vec2 features in clean and rain conditions.

%		Hot Anger	Irritation	Sadness	Panic Fear	Elation	Pleasure	distractors
clean	Hot Anger	**66.7**	3.3	0.0	6.7	6.7	0.0	16.7
	Irritation	13.3	36.7	3.3	3.3	0.0	20.0	23.3
	Sadness	0.0	6.7	**66.7**	3.3	0.0	23.3	0.0
	Panic	10.0	3.3	6.7	33.3	0.0	40.0	6.7
	Elation	13.3	3.3	0.0	0.0	50.0	3.3	30.0
	Pleasure	0.0	3.3	23.3	26.7	6.7	16.7	23.3
precision		**64.5**	**64.7**	**66.7**	45.5	**78.9**	16.1	–
rain SNR -1	Hot Anger	30.0	13.3	3.3	13.3	26.7	3.3	10.0
	Irritation	10.0	23.3	20.0	6.7	6.7	30.0	3.3
	Sadness	0.0	10.0	**63.3**	3.3	3.3	16.7	3.3
	Panic	16.7	3.3	0.0	16.7	20.0	36.7	6.7
	Elation	33.3	0.0	3.3	6.7	36.7	16.7	3.3
	Pleasure	10.0	3.3	20.0	23.3	23.3	20.0	0.0
precision		30.0	43.8	**57.6**	23.8	31.4	16.2	–

The cases misclassified as distractors are given all together. Darker shadowing indicates higher percentage, values >50% are boldface.

As shown in the perceptual study (cf. Table 3), sadness is the emotion by far best classified in background noise, showing a recall comparable to the one achieved in clean background (cf. 63.3% for rain and 66.7% for clean in Table 6). The same way as for the listeners, an increase in the confusion attracted by sadness in background noise is shown for the ML classification, which is displayed by a decrease in the precision of sadness when recognised in noisy background (57.6%) w. r. t. the clean one (66.7%); cf. precision in Table 6. The most prominent confusion pattern towards sadness is displayed by the low aroused emotion pleasure in both backgrounds: in clean, 23.3% of samples from pleasure were misclassified as sadness; in rain noise, 20.0% (cf. clean and rain for sadness and pleasure in Table 6). Similarly as for perception, this confusion pattern is not particularly shown in the opposite direction, since the confusion towards pleasure affected rather emotions other than sadness, in particular panic, followed by irritation (cf. the column for pleasure in Table 6). The confusion pattern between panic and pleasure is shown in both directions for ML: 40.0% and 36.7% of panic samples were misclassified as pleasure; 26.7% and 23.3% of pleasure samples were misclassified as panic (cf. clean and rain noise, respectively, in Table 6). For perception, the confusion was shown only towards panic. Finally, the spread of the responses for hot anger in ML classification, especially in background noise, is due to arousal-related confusion patterns: 13.3% of samples from hot anger were misclassified as panic, 26.7% as elation—a confusion pattern also shown in the opposite direction: 16.7% of samples from panic were misclassified as hot anger, 33.3% for elation (cf. hot anger, panic, and elation in Table 6). This can be seen more clearly in the NMDS, cf. Fig 7(b), by the overlap between hot anger and elation in background noise.

### Human vs machine: Assessing the two worlds

**Fig 8:**
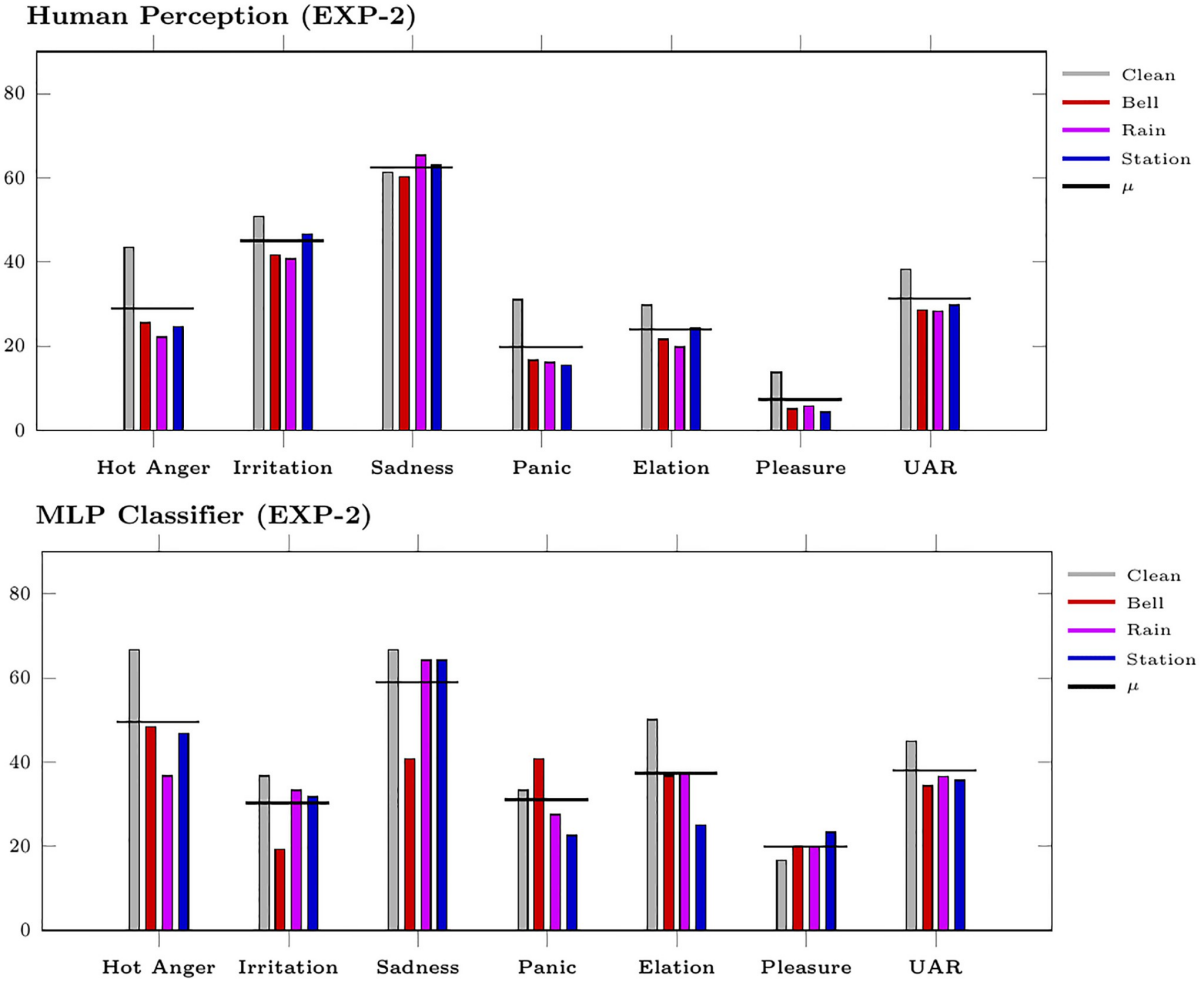
Recall per class and UAR (in%) for human perception and MLP classifier. The MLP is trained on data group D with wav2vec2 features. Results are given on EXP-2 considering all SNRs together for the noisy conditions. Mean across conditions (*μ*) is also given.

As the comparison of human vs machine yields similar results for EXP-1 [18] and EXP-2, we only report it for EXP-2. In Fig 8, the perceptual and classification outcomes are given, displaying for ML the best performing model, i. e., MLP with wav2vec2 features trained with data group D. The average across all SNRs is reported for both perception and classification. Concerning the overall classification, for both perception and classification, a similar UAR is displayed across conditions: for humans, UAR = 38.3 in clean, 28.3≤ UAR ≤29.7 in background noise; for machines, UAR = 45.0 in clean, 34.3≤ UAR ≤36.5 in background noise; cf. UAR in Fig 8. In clean condition, a similar trend for humans and machines can be observed (cf. clean in Fig 8): Sadness is the emotions best identified (61.3 for perception, 66.7 for classification), pleasure the worst (13.8 for perception, 16.7 for classification); all the others follow the same trend for humans and ML except for irritation, notably worse classified by machines than by humans (50.8 for perception, 36.7 for classification). This is due, as discussed in the previous section, by the confusion pattern between low aroused emotions.

These similarities between perception and automatic classification are more evident when looking at the NMDS. In Fig 7, we see that without background noise, the three ‘pillars’ in both constellations, i. e., for human perception (a) and MLP classification (b), are sadness, hot anger, and elation (cf. Clean in Fig 7). These emotional categories correspond to three distinct positions in the bi-dimensional space defined by Russel [20]: sadness shows a negative valence (left side) and low arousal (lower half); hot anger shows a negative valence (left side) and a high arousal (upper half); elation presents a positive valence (right side) and a high arousal (upper half). The distinction between these three emotions can also be mapped onto acoustic features, as observed in [69], in terms of minimum, mean, and maximum fundamental frequency (F0). The average across the six considered speakers is as follows: Sadness presents lowest values (99.3, 118.1, and 162.8 Hz), hot anger intermediate (255.1, 282.7, and 380.1 Hz), and elation highest (259.2, 293.5, and 419.2 Hz). Although these main pillars are clearly preserved for perception also in background noise, cf. Rain in (a), for classification, there is an overlap between hot anger and elation, which is due to the confusion between these two emotions. This arousal-related confusion is also shown, to some extent for panic, which is displayed closer to hot anger and elation in noise background than in the clean constellation, cf. Rain in (b).

The UAR decline linked to human perception and MLP classification in background noise (cf. UAR in Fig 8) goes along with an increment in confusion between emotions. This can be seen in the NMDS by a condensation of the emotions towards the centre for the background noise; cf. (a) and (b) for Rain in Fig 7. In the confusion matrices, this is indicated by lower precision as well as by lower recall (apart for sadness in perception and for pleasure in classification): cf. precision and recall (in the diagonals) for clean vs rain in Tables 3 and 6. Yet, especially for the basic emotions anger, sadness, and elation, the ‘cognitive-emotional space’ displayed for human perception and mirrored to some extent in the MLP classification of clean speech is—despite the condensation—still preserved in background noise. This is shown by the similarities between the clean constellation and the noisy one for both humans and MLP: cf. clean vs rain in Fig 7(a) for human perception and in Fig 7(b) for MLP classification. However, the instability of the ‘weaker’ emotions can be seen for irritation, panicked fear, and pleasure that are found towards the center and change places for humans and MLP, as seen in Fig 7(a) vs 7(b).

For evaluating the overall results, the average recall for each emotion across conditions (clean and noisy) is also given (cf. *μ* in Fig 8). When considering all the conditions together, the trend described for the recognition of clean samples generally persists: Sadness is clearly the emotion best recognised (on average 62.4% for humans, 59.0% for the MLP), pleasure the worst (on average 7.2% for humans, 20.0% for the MLP). Unlike for human perception (44.9%), the MLP classification shows a much lower average recall for irritation (30.2%). In contrast, the average recall for hot anger is much lower for human perception (28.9%) than for MLP (49.6%). We might speculate that in the case of hot anger, the paradoxical phenomenon reported in [70] is observed: peak emotions can be maximally ambiguous for humans; this could explain the lower performance for hot anger and the confusion with irritation, see Table 3. Obviously, this does not hold for machines.

## Discussion and limitations

In this section, we want to take up the metaphor of the five worlds sketched in the introduction summarising the results and discussing limitations of our approach:

**(i) The fuzzy world**: The NMDS solutions in Fig 7 demonstrate that the classes used can be mapped onto dimensions—but, especially for the noisy condition, only for the three ‘pillar’ categories hot anger, elation, and sadness, and not the same way for perception and ML. This surely relates both to the acoustic ambiguities mentioned passim and by that, to the limitations of a uni-modal modelling, and especially to the fact that valence is rather indicated with linguistic means. This is a limitation of our approach, due to the choice of keeping as many things equal as possible. What we can conjecture is that the ML confusions for the ‘weaker’ categories might be based on other criteria than the perceptual ones because it can be seen in Fig 7 that panic and irritation sort of change places—and do not really represent their ‘proper’ position on a valence dimension. Reassuring is that the ‘pillars’, i. e., the basic emotions anger, elation, and sadness, can really be found at the proper positions in the two-dimensional arousal/valence space, especially for clean speech.
**(ii) The closed world**: We attempted to model a more **open world**, i. e., a more realistic scenario where a larger set of emotional classes are known but not explicitly modelled. A fully realistic approach was, of course, not possible; yet, to the best of our knowledge, this procedure has never been adopted so far in the studies comparing perception and classification [39, 40, 62]. Our results show that **emulating more realistic conditions by introducing emotional distractors impairs the performance of both humans and machines in a similar way**. This is particularly relevant considering the difference in performance demonstrated for the ML models when using distractors, which shows that ML is definitely sensitive to confounding classes (which in real-life scenarios do exist but are not considered when validating SER systems).
**(iii) The clean world**: In order to assess how *real-life* acoustic pollution affects humans’ and machines’ recognition of emotions from speech, we also introduced a **noisy world**. This was done by considering a variety of noises and SNRs from real scenarios. It turned out that **regardless of type (real or artificial), noise impairs human perception and automatic classification of emotions in speech, increasing the confusion patterns towards low aroused emotions**. Perceptual and ML results show a similar trend: Noise conditions are affecting similarly, emotions in clean condition are being the best recognised.
**(iv) The small world**: Due to the difficulties typically associated to the collection and annotation of data for SER, we considered a **bigger world**: We assessed to which extent the performance differences between *‘traditional’ methods and state-of-the-art* procedures were affected by differences in the size of the training data. To carry out a fair comparison, the optimisation of the considered models for both the traditional and state-of-the-art feature sets was based on fixed set-ups. In our experiments, the **state-of-the-art feature representations employed, i. e.**, **wav2vec2** **embeddings, are trained on large external emotional databases and show an overall better performance than hand-engineered traditional audio features**. Yet, the disadvantage of wav2vec2 with respect to ComParE is its lack of interpretability. Furthermore, as shown in [34], our results confirm that the size of the training set influences the performance of ML models as well. This might be compared to humans’ capability to identify emotions in speech according to their developmental stage [24]: More years of experience (emulated by a bigger training set) yield better performance.
**(v) The one world**: Finally, we investigated **the two worlds**, i. e., we performed—for the first time—a one-to-one comparison between humans and ML on the same SER task. Our results show that, when guaranteeing comparable conditions, **similarities between human and ML on SER can be observed for strong emotion categories; weaker categories, however, seem to be handled differently**. This makes it likely that the performance of SER systems modelling a few controlled classes in unrealistic scenarios would hardly mirror human-like emotion recognition. Partially confirming the findings of [40], we showed that sadness is classified better than all other emotions (except hot anger in clean background) by the ML system; yet, in contrast to [40], our study confirmed this for listeners’ perception as well. Finally, one limitation of our study worth to be mentioned is that within a strictly controlled design, it might be impossible not to use acted nonsense speech.

## Conclusion

In this study, we have tried to address four fallacies typical of traditional affective computing research, which we have introduced with the metaphor of the four worlds: the ‘closed world’, the ‘clean world’, the ‘small world’ and the ‘one world’. By investigating first the ‘closed’, ‘clean’, and ‘small’ worlds, we were able to evaluate the impact of emotional distractors, environmental noise, and framework specificities (feature representations as well as architectures) in both human perception and ML classification of emotional speech. Through these three experiments, we systematically assessed the impact of distractor labels, noisy conditions, and ML aspects (in particular training size and features), which enabled us to define a more fair set-up. Finally, after the optimal ML set-up was identified, its performance was evaluated for the clean and most noisy condition in comparison with the perceptual results. Since the previous knowledge of a human might be, to some extent, comparable to the knowledge of the ML model acquired by its training on large scale data with distractor labels and noises, the presented experiments enabled us to perform a systematic one-to-one comparison between humans and machines, i. e., addressing for the first time the ‘one world’ fallacy.

From our experiments, which tried to emulate a more realistic setup than the one typically modelled in traditional affective computing, we can say that distractors impair both human perception and ML classification in a similar way. This is shown especially for sadness, i. e., the emotion best identified by humans and ML (showing a recall in clean conditions of 61.3% and 66.7%, respectively); this holds in background noise as well (under the strongest noise, i. e., rain at -1 dB SNR, sadness shows a recall of 65.7% and 63.3% for perception and ML, respectively). Besides the role of distractors in creating confusion amongst emotional categories, the parallels between perception and classification is also observed in general for the impact of noise: Considering all the SNRs together, the UAR is comparable amongst noises for both perception (28.3≤ UAR ≤29.7) and ML (34.3≤ UAR ≤36.5). Furthermore, state-of-the-art methods such as wav2vec2, due to the large amount of data used in the model training, perform better than traditional representation, such as acoustic hand-engineered features for which no training data is required during feature extraction. In preliminary experiments, we also employed DL architectures, such as Long Short-Term Memory Recurrent Neural Networks, along with those herein discussed; however, these resulted in lower and unsystematic performance, most likely due to the low size of the training partitions. wav2vec2 can partly circumvent this problem because it is based on a very large dataset modelling the linguistics and the phonetics of spoken language. Obviously, this helps for sparse datasets as well. This is shown by our results, where on average, across conditions and architectures, the UAR achieved with wav2vec2 features was 31.4% while the one for the hand-engineered ComParE features was 25.2%. The high impact of using a large training set is further confirmed by the performance of wav2vec2 with SVM, yielding a marked difference (*p* = .017) in the UAR achieved with a small dataset (for data group A, UAR = 26.7%) with respect to a large one (for data group D, UAR = 35.8%).

We do not claim to have solved the riddles of all the constrained worlds addressed; yet, we hope to have contributed towards widening the scope. We believe that the use of procedures similar to the ones presented should be considered in the future in order to more adequately evaluate the real potential of an SER system, by this guaranteeing more felicitous human-computer interactions. A further task to be addressed is a deeper evaluation of the specific acoustic features that might be more suitable in mirroring the confusion patterns across emotions shown by listeners’ perception. Needless to say, uni-modal modelling has to be complemented by multi-modal modelling, and especially by linguistics; yet, this might be rather complex, if really controlled scenarios are targeted. In our uni-modal modelling, we concentrated on specific short-comings of emotion modelling, employing traditional concepts such as the big emotion classes and the two ‘big’ dimensions. Of course, eventually this has to be substituted with richer, more fine-grained models. It remains to be seen whether and how DL-based models can be optimised for the use of sparse training data.
